# Evaluating clinical heterogeneity in relapsing polychondritis through unsupervised cluster analysis

**DOI:** 10.1186/s13075-026-03857-z

**Published:** 2026-07-11

**Authors:** Wenping Pan, Liguo Yin, Lantao Wang, Qing Wang, Tingting Zhai, Jin Cao, Xia Wang, Hongsheng Sun

**Affiliations:** 1 Department of Rheumatology and Immunology, The First Affiliated Hospital of Shandong First Medical University & Shandong Provincial Qianfoshan Hospital, Jinan, Shandong China; 2 Department of Rheumatology and Immunology, Shandong Provincial Hospital Affiliated to Shandong First Medical University, Jinan, China; 3 School of Public Health, Shandong university, Jinan, China

**Keywords:** Relapsing polychondritis, Cluster analysis, Prognosis

## Abstract

**Background:**

As a rare autoimmune disease, relapsing polychondritis (RP) exhibits individual variations in clinical manifestations, treatment response and prognosis. To date, no biomarkers have been implemented to clinical practice.This study aimed to apply cluster analysis to identify the clinical phenotype, clarify their prognosis predictor, and monitor treatment decisions to improve the outcomes of patients.

**Methods:**

A total of 135 RP patients hospitalized in two centers from January 2005 to December 2024 were involved in the study.The K-means clustering algorithm was used as the core clustering tool. The optimal number of clusters(K = 3)was determined through comprehensive evaluation using the Elbow Method and Silhouette Analysis.

**Results:**

Cluster analysis identified 3 RP phenotypes.Cluster 1 comprised 45 cases(33.3%),characterized by a hyperinflammatory state and predominant respiratory involvement; Cluster 2 included 11 cases (8.1%),defined by severe and multi-organ involvement Cluster 3 which accounted for 79 cases (58.52%), more than half of the cohort, was predominantly male and exhibited the most favorable prognosis.In contrast, Clusters 1 and 2 were associated with the highest rates of clinical deterioration.Conclusions: This study confirms that relapsing polychondritis is not a single-dimensional disease, but a clinical syndrome with significant heterogeneity. Random forest analysis further identifies that systemic inflammation markers (CRP, CAR, ESR) and sensory organ involvement (hearing impairment and inner ear dysfunction) as the most critical driving factors for distinguishing these subtypes.

## Introduction

Relapsing polychondritis (RP) is a rare systemic autoimmune disease characterized by relapsing episodes of inflammation and degeneration of cartilaginous tissues [1]. Its incidence ranges from 0.71 to 3.5 per million person-years [2–4].Typical clinical features include inflammation and destruction of nasal bridge, auricular cartilage, proteoglycan rich structures of the eyes and respiratory tract.Due to its rarity and the non-specific clinical manifestations, patients often experience significant diagnostic delay following symptom onset.Currently, no specific biomarkers are routinely used in clinical practice. The etiology and pathogenesis of RP remains unclear.Various factors have been implicated, including genetic susceptibility, autoimmune responses targeting cartilaginous structures, and dysregulation of cytokine and chemokine profiles that alter immune cell activation [5]. Thus, treatment is currently based on symptom control typically using steroids and immuno- suppressive drugs.A standard treatment strategy is lacking and management mainly relies on the clinician’s experience.The disease follows a progressive course, potentially leading to permanent cartilage destruction. Overall, survival outcomes for RP have improved considerably ‌over the past 10 years due to the availability of interventional treatments.However, the mortality rate remains high, with a standardized mortality ratio of 2.16 compared to the general population [2].

RP patients exhibit marked interindividual heterogeneity in clinical manifestation, disease progression and treatment response [1]. Moreover, growing evidence highlights significant variations in clinical phenotypes and prognostic outcomes across different ethnicities and geographic regions [6–8].These observations underscore the critical need for personalized management strategies. Refining the phenotypic classification of RP could yield substantial clinical benefits, yet there remains a notable lack of standardized frameworks for subtype stratification based on core clinical variables.

Clustering analysis, a powerful and widely employed data-mining tool in biological research, plays an important role in identifying patterns in complex diseases.It is also used to investigate novel or unexpected relationships that are not initially obvious.Furthermore, it can be used to identify subgroups of patients with similar trajectories in heterogeneous medical conditions, as it offers valuable insights into the mechanisms underlying disease heterogeneity.Therefore, in this study, we employ clustering analysis to investigate the existence of clinically significant phenotypes within a relatively large cohort of patients with RP.This analytical framework can be used to inform treatment decisions and improve clinical outcomes for RP patients, and thus contribute to the development of more personalized and effective care strategies.

## Methods

### Study population and data collection

A total of 135 patients with relapsing polychondritis (RP) were enrolled in this retrospective cohort study. These patients were recruited from two tertiary referral centers—the First Affiliated Hospital of Shandong First Medical University and the Affiliated Provincial Hospital of Shandong First Medical University—between January 2005 and December 2024. All patients met either McAdams or Damiani’s diagnostic criteria for RP [8], and written informed consent was obtained from each patient prior to study initiation. The clinical information of patients were collected.

Demographic and clinical characteristics of the 135 RP patients were systematically recorded.Clinical signs and symptoms related to RP were confirmed through a combination of examinations and imaging studies. In addition to routine laboratory test results, we collected data on three emerging inflammatory markers: the neutrophil-to-lymphocyte ratio (NLR), C-reactive protein-to-albumin ratio (CAR), and platelet-to-lymphocyte ratio (PLR) [9, 10]. At the time of initial treatment, all patients were evaluated by a specialized research team with expertise in RP management. Each patient underwent a standardized comprehensive assessment, which included laboratory investigations, laryngeal imaging, and chest imaging. Disease severity at presentation was quantified using the Relapsing Polychondritis Disease Activity Index (RPDAI ) [11].

### Cluster analysis

To maximize the sample size and ensure the robustness of the analysis, missing data were imputed using the mean imputation method. Variables with zero variance were excluded. To eliminate the influence of dimensional differences across various indicators (e.g., age and C-reactive protein [CRP]), all continuous variables were subjected Z-score normalization.

The K-means clustering algorithm was adopted as the core classification tool. Patients with similar clinical characteristics were grouped into the same cluster by calculating the Euclidean distance between samples. The optimal number of clusters, K = 3, was determined through a comprehensive evaluation combining the Elbow Method and Silhouette Analysis. To validate the clustering performance, Principal Component Analysis (PCA) was utilized to reduce the dimensionality of the multi-dimensional data and visualize the results.

## Result

### Characteristics of cohort

This study included 71 male and 64 female patients with an average age of 51.21 ± 14.65 years at disease onset(IQR 43–63). The average time from initial symptom onset to diagnosis was 9.68 *+* 16.25 months (IQR 2–10 ). About thirteen clinical variables were collected at enrolment, including demographic characteristics (age, sex), initial organ involvement (ear, nose, respiratory tract, joints, etc.), and laboratory markers (ESR, CRP, NLR, CAR, etc.).Detailed clinical characteristics are shown in Tables 1 and 2.

**Table 1:** Demographics and clinical features

Demographics	Total	Cluster 1	Cluster 2	Cluster 3	*P*
N(%)	135	45(33.33)	11(8.15)	79(58.52)	
Sex/female(n,%)	64(47.41)	22	8	41	
Duration(year)	6.49 ± 4.49	5.34 ± 4.27	5.12 ± 4.20	7.33 ± 4.52	
Mean delay duration (month)	9.68 *+* 16.25	8.33 ± 14.62	15.45 ± 20.98	9.64 ± 16.46	
Age(mean ± SD)(year)	51.21 ± 14.65	53.82 ± 14.35	60.18 ± 10.73	48.47 ± 14.66	< 0.01
Organ involvement (n,%)					
Auricular chondritis	57(42.22)	7	7	43	
Tracheobronchitis	52(38.52)	28	4	20	
Laryngotracheitis	27(20.0)	16	4	7	
Articular involvement	25(18.52)	13	2	10	
Hearing impairment	18(13.33)	3	11	4	
Nasal Chondritis	18(13.33)	9	5	4	
Costal cartilages	14(10.37)	13	1	0	
Ocular involvement	14(10.37)	1	4	9	
Inner ear	11(8.15)	2	8	1	
Cutaneous lesions	2(1.48)	1	0	1	
Systemic involvements					
Interstitial pneumonia/pleuritis	34(25.19)	9	6	19	
Fever	26(19.26)	21	2	3	
Anemia	17(12.59)	11	5	1	
Liver	15(11.11)	10	2	3	
Neurological involvement	5(3.7)	0	3	2	
Kidney	2(1.48)	0	2	0	
Cardiovascular involvement	1(0.74)	0	1	0	
Tracheotomy	6(4.44)	5	1	0	
Mortality	13(9.63)	5	8	0	

**Table 2:** Laboratory indicators and other parameters

Laboratory data (mean *+* SD)	Total	Proportion of missing variables	Cluster 1	Cluster 2	Cluster 3	*P*
ESR(mm/h)	44.33 ± 33.37		68.89 ± 31.58	52.18 ± 23.99	29.25 ± 26.25	< 0.001
CRP(mg/L)	39.31 ± 47.39		85.59 ± 53.08	40.41 ± 30.51	13.37 ± 16.34	< 0.001
ALB(g/L)	37.87 ± 5.46	3.71%(5)	32.75 ± 5.72	34.51 ± 4.18	40.89 ± 4.14	< 0.001
NLR	5.24 ± 5.95		7.74 ± 8.47	8.06 ± 8.70	3.54 ± 2.28	< 0.001
PLR	208.69 ± 140.37		302.92 ± 173.73	252.16 ± 158.20	160.03 ± 90.48	< 0.001
CAR	1.81 ± 1.97	3.71%(5)	3.34 ± 2.30	1.24 ± 1.12	0.40 ± 0.46	< 0.001
Activity Scores						
RPDAI	22.93 ± 10.93		26.42 ± 9.93	40.55 ± 11.00	18.49 ± 7.96	< 0.001
Treatment						
Adequate steroids	64(47.41%)		23	7	25	
Moderate steroids	62(45.93%)		19	3	49	
Extra high steroids	9(6.67%)		3	1	5	
CTX	28(20.74%)		14	5	9	
MMF	28(20.74%)		5	6	17	
MTX	52(38.52%)		16	4	32	
LEF	10(7.41%)		8	0	2	
AZA	5(3.70%)		2	0	3	
JAKi	5(3.70%)		2	0	3	
TPT	12(8.89%)		2	0	10	
TNF-αi	8(5.93%)		4	1	3	
IL-6i	7(5.19%)		3	2	2	

There were 2 missing data of ALB and CAR in Cluster 1,3 missing data ALB and CAR in Cluster 3

### Cluster analysis

By combining ANOVA variance analysis and random forest feature importance ranking, several clinical features were identified as the most significant determinants of cluster membership.A 3 cluster model was found to best fit the overall data set, with no significant gender differences observed among the three clusters.

Cluster 1 describes a cohort with respiratory involvement and hyperinflammation phenotype (*n* = 45,33.33%). These patients exhibited a high incidence rate of laryngeal and primary bronchial involvement, affecting 59.26% and 53.85% of the cluster population respectively.As shown in Table 1,Cluster 1 had the highest percentage of patients who underwent tracheotomy (11.11%). Fever was more common in this cluster, with a significantly higher incidence rate compared to the other two clusters. Additionally, three parameters—ESR, CRP, and PLR showed statistically significant differences across clusters.The core characteristic of these patients is an active systemic inflammatory response, as indicated by the highest CAR (2.97 ± 1.97),a composite index reflecting both inflammation and nutritional indicators. Cluster 1 patients are in critical condition, characterized by a high risk of airway collapse and severe systemic inflammatory storm.

Cluster 2 is defined as a severe phenotype with sensory and multi-system involvement (*n* = 11,8.15%). With an average age of 60.18 years, this cluster included the oldest patients among the three groups. Nerve damage was present in 27.27% of patients, and all patients in this cluster had hearing impairment.Renal impairment was observed in 18.18% of cases, while ocular and nasal involvement rates were the highest across all clusters. Anemia of chronic disease was detected in 45.45% of Cluster 2 patients. Although this cluster had the smallest sample size (11 patients, 8.15% of the total cohort), it was characterized by the highest Disease Activity Score (RPDAI) and a high mortality risk.

Cluster 3 included the largest proportion of patients, with 79 cases accounting for nearly 58.52% of the cohort. We describe this cluster as auricular involvement and mild phenotype.Symptoms in these patients are localized, primarily presenting with classic auricular onset (54.43%), which facilitates early diagnosis.The involvement of internal organs (heart, liver, kidneys, nerves) and the respiratory tract was relatively rare. Overall, these patients had a milder clinical course, with the lowest RPDAI score (18.49) and ESR level (29.25 mm/h). Cluster 3 represents the most typical clinical manifestation of RP, with mild systemic damage and a relatively favorable prognosis.

### Clinical variables of the three clusters

**Fig. 1:**
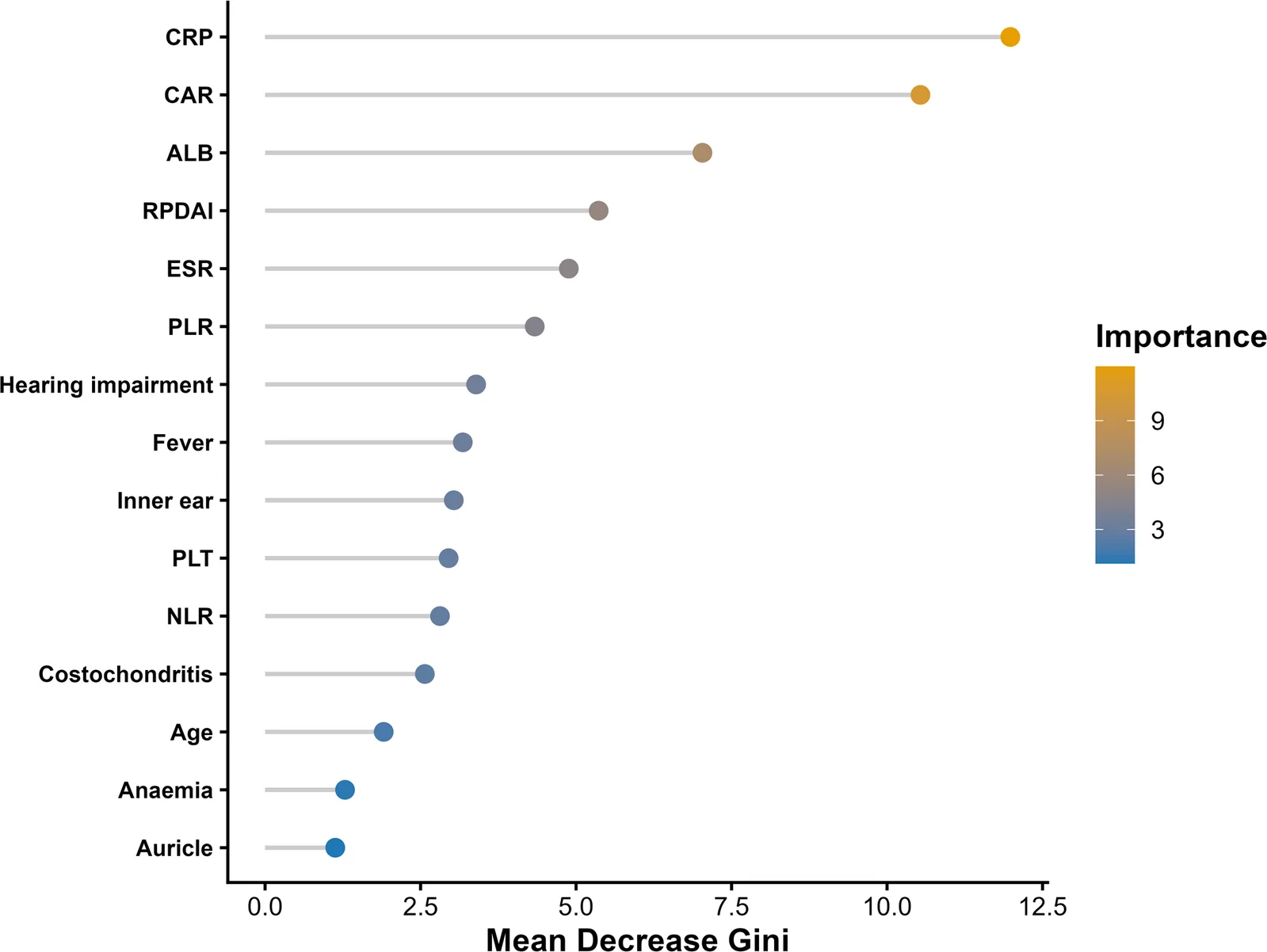
Key indicators for cluster. 15 key indicators for clustering algorithm were presented. The top three parameters were CRP, CAR and ALB

**Fig. 2:**
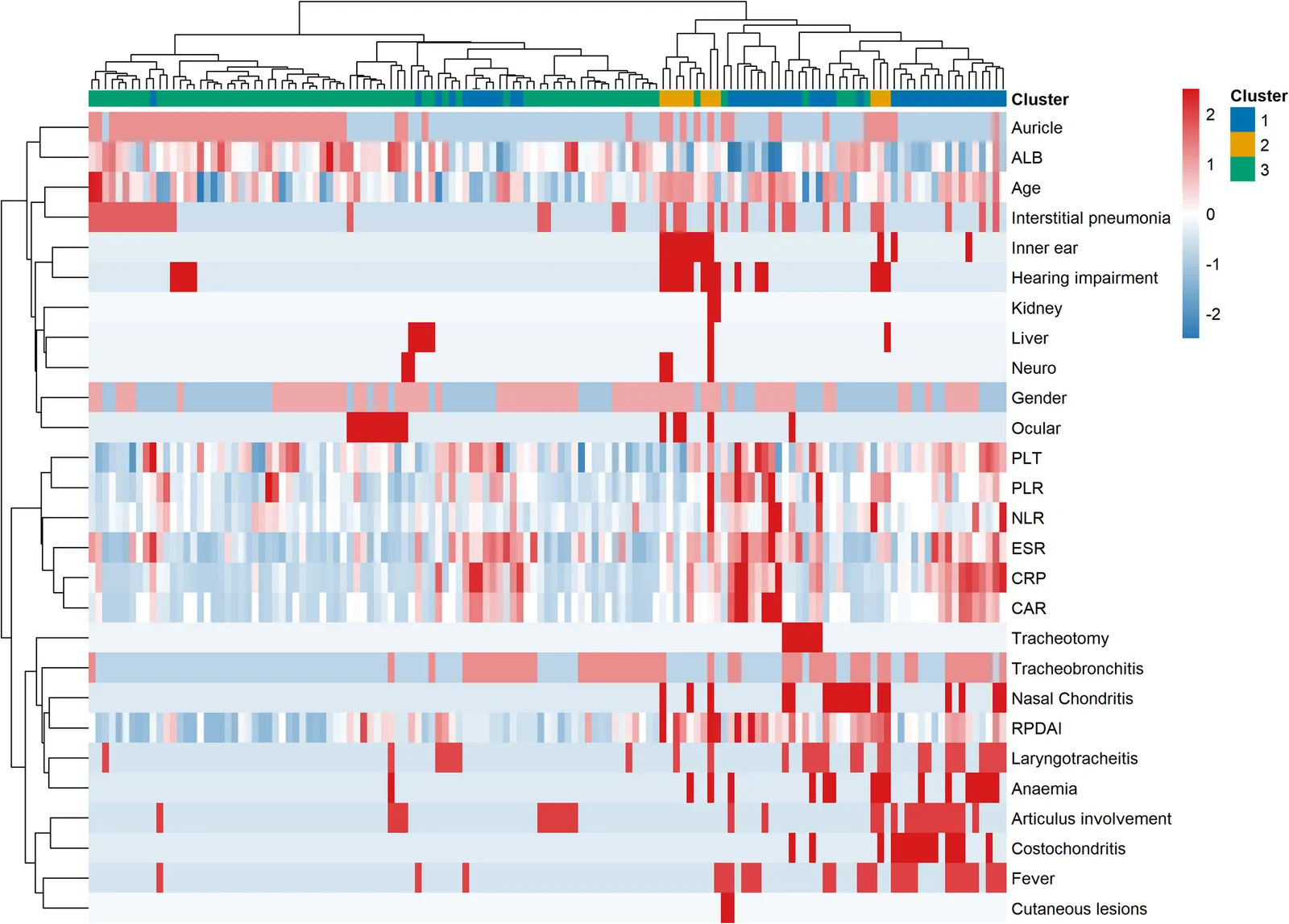
Heat map of the cluster feature variables. Heatmap displaying z-score normalized values of 27 clinical variables (rows) across 135 patients (columns). Both rows and columns are ordered by hierarchical clustering (Ward’s linkage). The color bar at the top indicates cluster assignment: Cluster 1 (blue, *n* = 45), Cluster 2 (yellow, *n* = 11), and Cluster 3 (green, *n* = 79). Red indicates values above the cohort mean; blue indicates values below the mean. Row clustering reveals three major feature groups: (i) auricular and systemic features (Auricle, Inner ear, Hearing impairment, ALB); (ii) inflammatory markers (CRP, ESR, NLR, PLR, CAR); and (iii) airway and cartilaginous involvement features

**Fig. 3:**
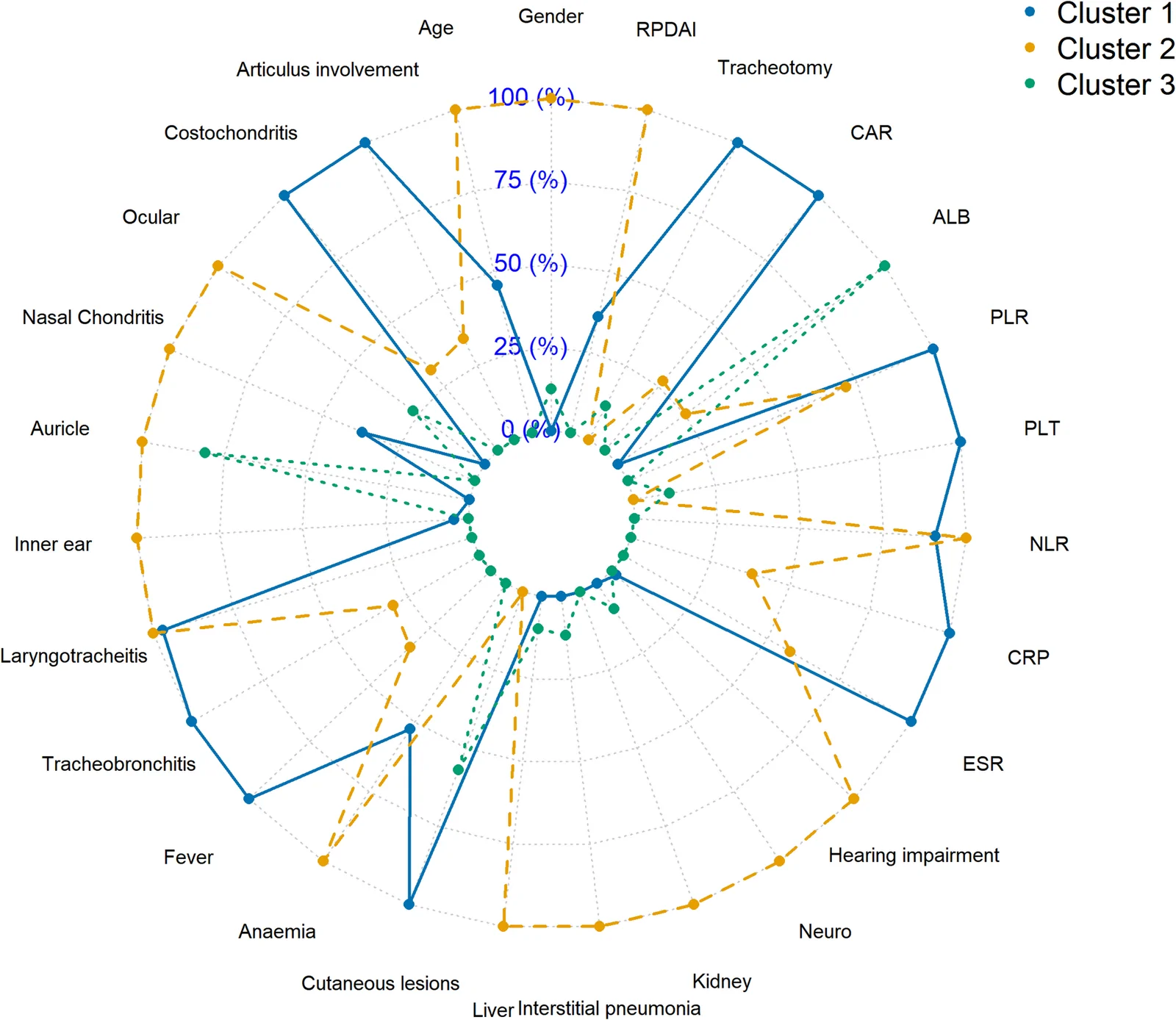
The radar charts of the cluster feature variables. The radar chart represented the multidimensional distribution characteristics of three Clusters across all included features. The scale of each axis in the chart was the relative position after inter-group relative normalization, with the maximum mean value of the three Clusters on that variable corresponding to 100% and the minimum corresponding to 0%. The mean values of each Cluster were linearly mapped within this range. This chart showed the relative high and low relationships of each feature among Clusters

Key features are crucial for clustering.Clinical variables were ranked according to their importance.(Fig. 1) Heat maps of cluster feature variables and radar charts illustrate the distinct performance of each variable across the identified clusters (Figs. 2 and 3).Notably, inflammatory indicators such as CRP and CAR, appear to play a more important role in clustering than clinical symptoms. The Relapsing Polychondritis Disease Activity Index (RPDAI) is a valuable tool for assessing disease activity, as it comprehensively captures all aspects of disease activity through a combination of typical clinical symptoms and laboratory parameters.Previous studies have reported that novel inflammatory markers, including C-reactive protein to albumin ratio (CAR)and the platelet-to-lymphocyte ratio (PLR), are associated with RPDAI scores [9, 10]. These two emerging inflammatory indexes, along with other relevant features, constitute key factors influencing clustering performance. A significant positive correlation was found between PLR, NLR, CAR and RPDAI(*R* = 0.301,0.227and 0.278). In contrast, a significant negative correlation was found between airway involvement and auricular chondritis.(*R*= -0.487,*p* < 0.001).

**Fig. 4:**
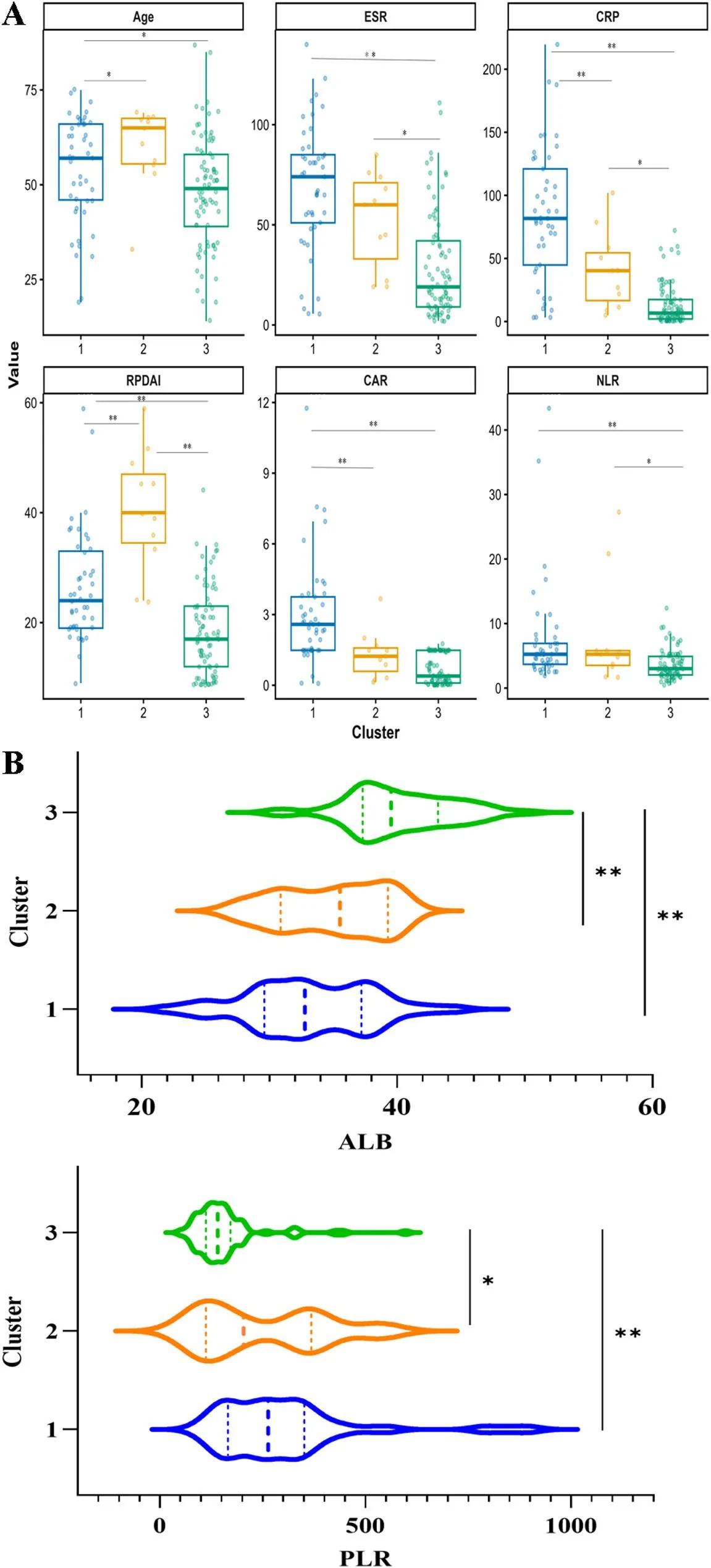
Comparison of vital indicators among three clusters. **A **Continuous variables were compared among three groups using the Kruskal–Wallis test. There existed overall distribution significant difference among three Clusters in these indicators.Pairwise comparisons were performed using Dunn’s Test. * *p* < 0.05;** *p* < 0.001. **B **One-way analysis of variance (ANOVA) showed statistically significant differences in albumin levels and PLR across the three clusters* *p* < 0.05༛** *p* < 0.001

Continuous variables were compared among three clusters using the Kruskal -Wallis test.The results revealed statistically significant differences in age, ESR, CRP, CAR, NLR, and RPDAI among the three clusters (Fig. 4A) Additionally, one-way analysis of variance (ANOVA) showed statistically significant differences in albumin levels and PLR across the three clusters. (Fig. 4B)

### Principal component analysis of the clusters

**Fig. 5:**
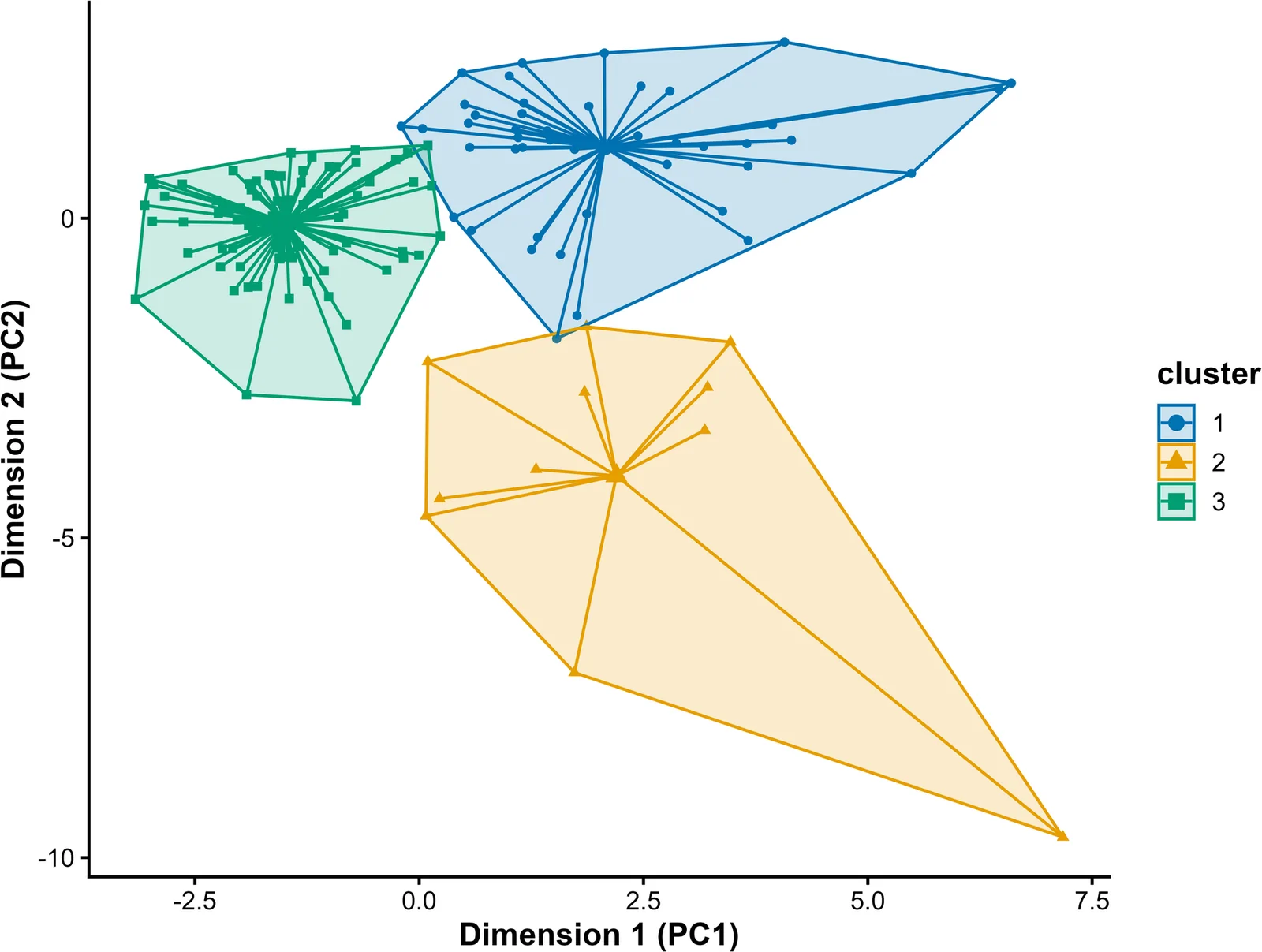
Principal component analysis of the clusters. PC1 and PC2 are not specific clinical indicators, but rather the comprehensive reflection of multiple variables weighted according to certain weights. They represent the most significant structural differences within the data.This PCA plot illustrated the distribution structure of the three clusters in a two-dimensional space. If the three clusters of points showed significant separation in PC1 and PC2 space, it indicated that these clusters exhibit significant differences in multidimensional clinical characteristics

The core idea of Principal Component Analysis(PCA) is to transform multiple original clinical variables into a small set of new composite indicators via linear combinations, termed “Principal Components”. Firstly, 29 clinical variables are selected.Then, 2 zero-variance variables are removed.Finally, 27 variables are used for PCA analysis.The first principal component (PC1) captures the direction of of maximum variance in the original data, while the second principal component (PC2) accounts for the next largest source of variation, under the constraint of orthogonality to PC1 (i.e., statistical independence from PC1).We employed PCA to identify which clinical variables drove the observed clustering patterns.(Fig. 5).

**Fig. 6:**
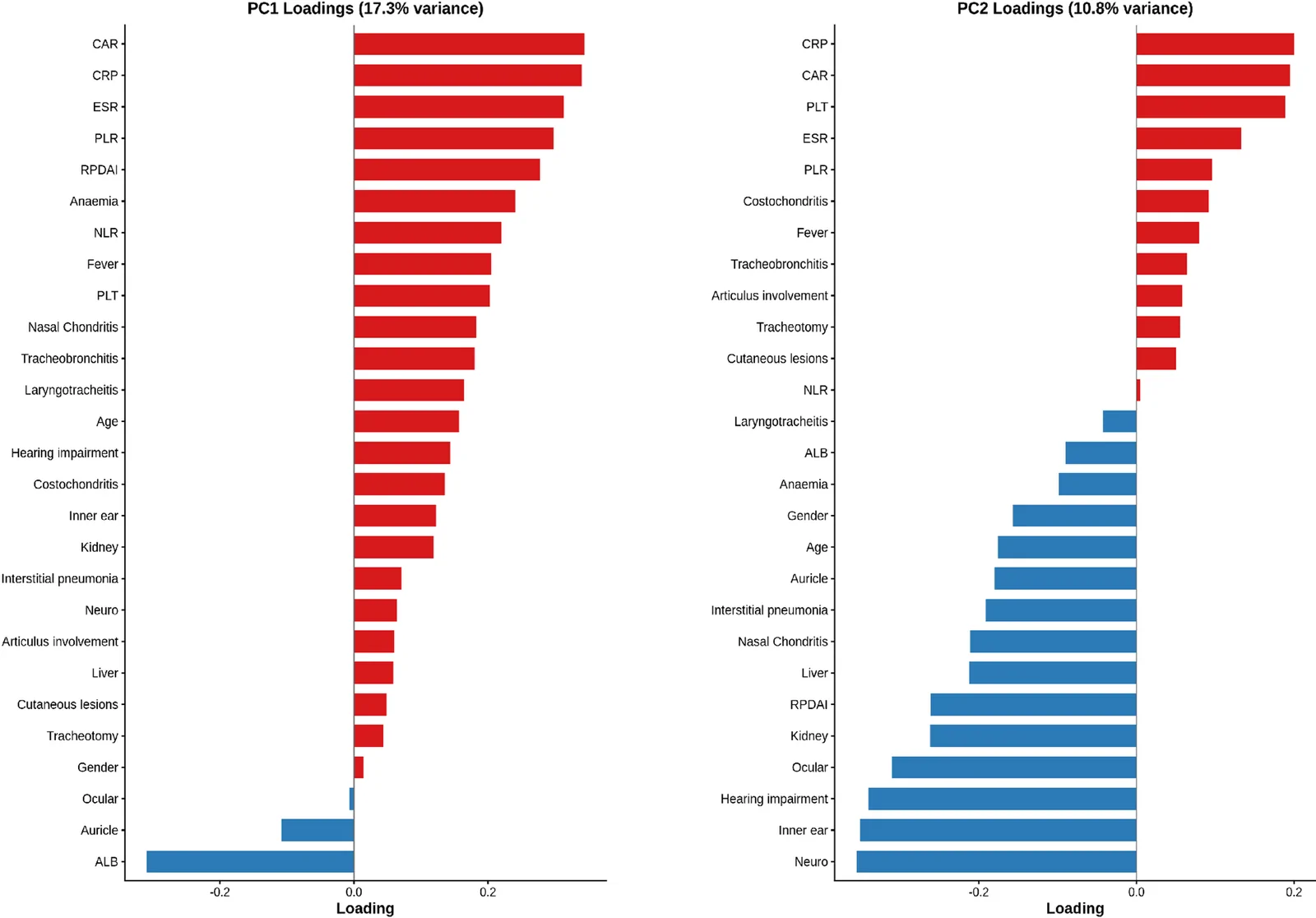
Variables loading for PC1 and PC2. PC1:CAR (+ 0.344), CRP (+ 0.340), ESR (+ 0.313), PLR (+ 0.298), and RPDAI (+ 0.277) have significant positive loading, while ALB has significant negative loading (-0.309).PC2:Neurological involvement (-0.355), inner ear involvement (-0.351), hearing impairment (-0.340), eye involvement (-0.310), kidney involvement (-0.261), and RPDAI (-0.261) have significant negative loading. Meanwhile, CRP, CAR, PLT, and ESR have relatively large positive loads on PC2

PC1 accounts for 17.3% of the total variance, and the variables with high absolute loading are variables related to systemic inflammation and disease activity. PC2 accounts for 10.8% of the total variance. Within the PCA framework, shifts along the PC1 axis correspond to changes in the overall inflammatory intensity of patients. In contrast, PC2 primarily captures differences in organ involvement patterns. Variable loading for PC1 and PC2 were shown in Fig. 6.

### Treatments of three Cluster

Systemic glucocorticoids are the first line treatment to RP.At the initiation of treatment, most patients received adequate(1 mg/kg/day prednisone equivalent)or moderate( 0.5 mg/kg/day prednisone equivalent) doses of glucocorticoids.(47.41% and 45.93% respectively)Only 9 patients received an excessively high glucocorticoids (> 1 mg/kg ≦ 200 mg/day prednisone equivalent). Small numbers of patients(9) did not administer immunosuppressants.The group received the following immuno- suppressants in sequence: methotrexate(38.52%), cyclophosphamide (20.74%), mycophenolate mofetil(20.74%), tripterygium glycosides (8.89%),leflunomide (7.41%), azathioprine(3.70%) and Janus kinase inhibitor(3.70%).‌ Biologic therapeutic agents such as etanercept(tumor necrosis factor α inhibitors) and tocilizumab (IL-6 inhibitors) had been introduced in patients who failed to the routine treatment. High-dose glucocorticoids and intensive immunosuppressive therapy were predominantly administered Cluster 1 and Cluster 2.Details are shown in Table 2.

## Discussion

Cluster analysis is an essential tool for gaining insight into disease heterogeneity and has been used to identify distinct clinical phenotypes with similar traits. This method provides a feasible approach to stratify conditions with high clinical variability, such as asthma, diabetes mellitus, and interstitial lung disease [12–14].The identification of disease phenotypes based on clinical presentation helps guide precise management strategies.To the best of our knowledge, only two main clinical patterns have been described in RP so far.In 2016, French researchers [15]identified 3 clinical phenotypes: the “hematologic”, “respiratory” and “mild” phenotypes based on the data from a cohort of 142 patients.The largest group, comprising 65% of patients, corresponded to the mild form, which was associated with an absence of severe complications and a low recurrence risk.The respiratory phenotype, accounted for 26% of cases, was characterized by younger patients with predominant airway(tracheobronchial or laryngeal)involvement.This phenotype associated with restricted respiratory function and a high infection rate.The hematologic phenotype, observed in 9% of patients, was characterized by older age and a diagnosis of myelodysplastic syndrome.It was the most severe phenotype, with a mortality rate of 58%.However, in our patient cohort, only one patient had a hematological comorbidity (multiple myeloma).Indeed, previous series [15–18] have demonstrated that hematological diseases are uncommon in RP.The hematologic subgroup in Dion’s [15] research exhibited characteristics resembling those of patients with the newly identified disease, VEXAS syndrome.The discovery of VEXAS syndrome in 2020 could explain the association between relapsing polychondritis and myelodysplastic syndromes in some patients [19]. VEXAS syndrome should be regarded as a disease mimicker, rather than as a distinct subtype of relapsing polychondritis. Detection of *UBA1* mutations should be strongly considered for elderly (> 50 years of age)patients who had haematological abnormalities [20].Nevertheless,12.5% of patients in the present study had anemia of chronic inflammation.It was more common in Cluster 1 patients. Ferrada et al. identified three similar phenotypes [21]. A minority of patients, had a classic pattern of disease characterized by ear chondritis and extensive cartilage damage to nose and upper airway cartilages. A Majority of patients were defined by lower-airway predominant disease. A few patients presented with limited cartilaginous involvement and hematologic abnormalities .

Interestingly, researchers observed a negative correlation between airway involvement and auricular chondritis [16, 21]. Thereby, Shimizu [22] and Liu [16] developed: auricular pattern(A), respiratory pattern (R group) and Overlap pattern(O group) based on their different prominently tissue involvement and disease progression.The majority of patients belonged to A pattern and R pattern at disease onset, while O pattern was less common.A pattern was associated with the best prognosis and a relatively lower inflammatory state. Notably, their studies emphasized that clinical patterns may transform during follow-up.In our cohort, only 4.76% of patients exhibited both features at disease onset, corresponding to the Overlap pattern. We also observed a negative correlation between airway and auricula involvement, which were the two major initial manifestations of RP.

It is well known that RP is a systemic autoimmune disease, that can affect multiple organs.Relying solely on classifications based on airway and auricula involvement may not provide a comprehensive view of disease heterogeneity.Through unsupervised machine learning clustering analysis, we identified three distinct clinical phenotypes: respiratory-high inflammation type, sensory organ-involved multisystem severe type, and auricular-dominant mild type. It Provides a unique perspective on the classification of disease. Principal component analysis revealed that these clusters exhibited statistical differences in multidimensional clinical characteristics, validating them as non-arbitrary categorizations.Random forest analysis further revealed that systemic inflammation levels (CRP, CAR, ESR) and sensory organ involvement (hearing/inner ear dysfunction) are critical discriminators for these subtypes. Patients assigned to cluster 1 characterized by its extremely high airway involvement and heavy inflammatory burden.Despite having the highest CRP, PLR and CAR, the RPDAI was not very high due to fewer multiorgan involvement in this cluster.Given its high risk of airway obstruction, clinicians should recognize it as an early warning signal of “critical and emergency conditions”. We recommend early dynamic airway assessment (e.g. bronchoscopy) and consideration of more aggressive anti- inflammatory strategies (such as early biologic agents combination therapy) in treatment to prevent life-threatening airway collapse. Patients in Cluster 2 (multiple system/high-score type), though comprising a small patient subset, presented with the most complex clinical manifestations. Cluster 2 had the highest RPDAI because of multiple organs involvement. Atypical initial symptoms and widespread multiple organs involvement often lead to misdiagnosis and diagnostic delay. Cluster 2 exhibited the longest misdiagnosis period due to its non-specific clinical presentation.It is therefore crucial for clinicians, especially non-rheumatologist, to maintain high vigilance for this rare disease. We strongly recommend implementing a Multidisciplinary Team approach to address this challenge, with management priorities focused on preventing irreversible sensory organ damage and controlling severe disease activity.Cluster 3 (mild type) is the most common subtype, characterized by mild disease condition. These patients had the lowest RPDAI and presented excellent long-term survival.The treatment goal should primarily focus on reducing recurrences. Despite having mild disease, regular follow-ups are still necessary to monitor disease progression.

Patients in Cluster 1 and 2 received more intensive treatment, including high dose of glucocorticoid and strong immunosuppressants, which may increase the risk of infections.None of our patients died from airway collapse or obstruction due to RP.Infectious complications is the leading cause of mortality.Inappropriate corticosteroids and strong immunosuppressants are risk factors for severe infections.Administration of biologics, such as interleukin-6 inhibitors, could help reduce the dose of glucocorticoids and immunosuppressants.For elderly patients in Cluster 3, tripterygium glycosides offer as safer and milder alternative to conventional immunosuppressants. Overall, the classification system established in this study provides an evidence-based foundation for precision and stratified management of RP.

Predictive biomarkers are pivotal for individualized disease monitoring. Unfortunately, The rarity of relapsing polychondritis (RP) has hindered the development of the application of biomarkers in relapsing polychondritis (RP).In recent years, the value of composite indicators such as the Systemic Immune Inflammation Index (SII), Neutrophil-to-Lymphocyte Ratio ‌(NLR), platelet- to- lymphocyte ratio ‌(PLR), C-Reactive Protein to Albumin(ALB) Ratio (CAR) have gained increasing attention for their value in disease early warning and severity assessment [9, 10].During the acute-phase response, CRP levels are elevated by inflammatory mechanisms, whereas serum albumin levels are reduced.Cluster 1 and Cluster 2 in a high inflammatory state had lower serum albumin levels.CAR is one of sensitive composite biomarkers that integrate both nutritional condition and inflammation of the body. Both ALB and CAR existed statistical differences among three clusters.By integrating multiple blood biomarkers parameters such as platelets, neutrophils, and lymphocytes, these composite indicators provide a comprehensive reflection of the body’s inflammatory and immune status. Our study confirms that three new inflammatory markers(PLR, NLR and CAR) are important indicators for disease classification and exhibited strong correlations with RPDAI.Notably, these parameters are simple to measure and widely available in routine clinical practice, making them highly practical for assisting clinicians in patient stratification and the formulation of tailored treatment strategies.

### Limitations

Our study has several limitations. First, as a retrospective analysis of hospitalized patients, inherent selection bias may exist, as the cohort primarily includes individuals with relatively severe disease manifestations, potentially limiting the generalizability of our findings to milder or non-hospitalized cases. Second, recall bias cannot be excluded, as participants may have inaccurately reported past symptoms, disease onset timing, or exposure history, which could affect the reliability of data related to disease progression and risk factor associations.Third, the cross-sectional design of this study precludes the ability to establish temporal relationships between variables and assess long-term outcomes. Without longitudinal follow-up, we may have underestimated the frequency of organ involvement, particularly in patients with short disease courses, as certain complications or progressive organ damage may emerge over time rather than at the initial presentation. Future prospective, multicenter studies with larger sample sizes and extended follow-up periods are warranted to validate our findings and address these limitations.In addition, due to the small number of Cluster 2, the statistical efficiency is limited. The results of this study need to be validated in future multicenter, large sample cohorts.

## Conclusions

In summary, our study found that several biomarkers, including CAR, PLR and CRP are significantly associated with activity index in patients with relapsing polychondritis. These biomarkers play a critical role in patient stratification. Through unsupervised clustering analysis, we categorized RP patients into three distinct clinical phenotypes, each characterized by unique organ involvement patterns and prognostic trajectories. This classification system provides clinicians with a practical tool to rapidly assess individual patients’ prognostic risks, tailor treatment strategies, and ultimately optimize long-term quality of life for patients with RP.

## Data Availability

No datasets were generated or analysed during the current study.

## Abbreviations

ALB: Albumin
ANOVA: Analysis of Variance
AZA: Azathioprine
CAR: C-reactive protein to albumin ratio
CRP: C-reactive protein
CTX: Cyclophosphamide
ESR: Erythrocyte sedimentation rate‌
IQR: Interquartile Range
JAKi: Janus kinase inhibitor‌
LEF: Leflunomide
MMF: Mycophenolate Mofetil
MTX: Methotrexate
NLR: Neutrophil to lymphocyte ratio
PCA: Principal Component Analysis
PLR: Platelet to lymphocyte ratio
RP: Relapsing polychondritis
RPDAI: Relapsing Polychondritis Disease Activity Index
TPT: Tripterygium glycosides
TNF-αi: Tumor Necrosis Factor-alpha inhibitor‌
IL-6i: Interleukin-6 inhibitor
